# A clinicopathological study of non-functioning pituitary neuroendocrine tumours using the World Health Organization 2022 classification

**DOI:** 10.3389/fendo.2024.1368944

**Published:** 2024-05-02

**Authors:** Chariene Shao-Lin Woo, Ronnie Siu-Lun Ho, Grace Ho, Hoi-To Lau, Carol Ho-Yi Fong, Johnny Yau-Cheung Chang, Eunice Ka-Hong Leung, Lawrence Chi-Kin Tang, Ivan Kwok-Ming Ma, Alan Chun-Hong Lee, David Tak-Wai Lui, Yu-Cho Woo, Wing-Sun Chow, Gilberto Ka-Kit Leung, Kathryn Choon-Beng Tan, Karen Siu-Ling Lam, Chi-Ho Lee

**Affiliations:** ^1^ Department of Medicine, School of Clinical Medicine, Li Ka Shing (LKS) Faculty of Medicine, The University of Hong Kong, Hong Kong, Hong Kong SAR, China; ^2^ Department of Anatomical Pathology, Queen Mary Hospital, Hong Kong, Hong Kong SAR, China; ^3^ Department of Radiology, Queen Mary Hospital, Hong Kong, Hong Kong SAR, China; ^4^ Department of Surgery, School of Clinical Medicine, Li Ka Shing (LKS) Faculty of Medicine, The University of Hong Kong, Hong Kong, Hong Kong SAR, China

**Keywords:** non-functioning pituitary neuroendocrine tumours, 2022 WHO classification, pituitary adenoma, PitNET classification, transcription factors

## Abstract

**Background:**

The 2022 World Health Organization (WHO) classification of pituitary neuroendocrine tumour (PitNET) supersedes the previous one in 2017 and further consolidates the role of transcription factors (TF) in the diagnosis of PitNET. Here, we investigated the clinical utility of the 2022 WHO classification, as compared to that of 2017, in a cohort of patients with non-functioning PitNET (NF-PitNET).

**Methods:**

A total of 113 NF-PitNET patients who underwent resection between 2010 and 2021, and had follow-up at Queen Mary Hospital, Hong Kong, were recruited. Surgical specimens were re-stained for the three TF: steroidogenic factor (SF-1), T-box family member TBX19 (TPIT) and POU class 1 homeobox 1 (Pit-1). The associations of different NF-PitNET subtypes with tumour-related outcomes were evaluated by logistic and Cox regression analyses.

**Results:**

Based on the 2022 WHO classification, the majority of NF-PitNET was SF-1-lineage tumours (58.4%), followed by TPIT-lineage tumours (18.6%), tumours with no distinct lineage (16.8%) and Pit-1-lineage tumours (6.2%). Despite fewer entities than the 2017 classification, significant differences in disease-free survival were present amongst these four subtypes (Log-rank test p=0.003), specifically between SF-1-lineage PitNET and PitNET without distinct lineage (Log-rank test p<0.001). In multivariable Cox regression analysis, the subtype of PitNET without distinct lineage (HR 3.02, 95% CI 1.28-7.16, p=0.012), together with tumour volume (HR 1.04, 95% CI 1.01-1.07, p=0.017), were independent predictors of a composite of residual or recurrent disease.

**Conclusion:**

The 2022 WHO classification of PitNET is a clinically useful TF and lineage-based system for subtyping NF-PitNET with different tumour behaviour and prognosis.

## Introduction

Classification of pituitary neuroendocrine tumour (PitNET) has evolved since 2004, from a system based on haematoxylin and eosin (H&E) staining, immunohistochemical (IHC) staining of anterior pituitary hormones and clinical phenotype, to a classification employing the transcription factors that were first introduced in 2017 by the World Health Organization (WHO), and to the latest 2022 WHO classification where PitNET lineage forms the core of the classification (1). In the 2017 WHO classification, the use of transcription factor stains was advocated to complement traditional IHC staining of anterior pituitary hormones when classifying PitNET into subgroups based on their lineages. Accordingly, tumours with POU class 1 homeobox 1 (Pit-1)-positivity with IHC staining for growth hormone (GH), prolactin (PRL), or thyroid stimulating hormone (TSH) were classified as somatotroph tumours, lactrotroph tumours, or thyrotroph tumours, respectively. Corticotroph tumours were characterized by tumours stained positively for T-box family member TBX19 (TPIT), while gonadotroph tumours were characterized by positivity for steroidogenic factor (SF-1). PitNETs that were stained negatively for both pituitary hormones and transcription factors were known as null cell PitNETs, while plurihormonal and double/triple PitNETs were characterized by positivity for either more than one hormone or transcription factors (1). In the latest 2022 WHO classification, the role of transcription factor in classifying PitNET was further consolidated, and all PitNETs were grouped under four separate entities, namely Pit-1-lineage, TPIT-lineage, SF-1-lineage PitNETs, as well as PitNETs with no distinct cell lineage (2). The latter encompasses the null cell and double/triple PitNETs as defined in the 2017 WHO classification. This shift in nomenclature from “hormone-producing adenoma” to “-trophic tumours”, and to the latest use of “-lineage PitNET” highlights the importance of transcription factors in cell differentiation and regulation (3).

Non-functioning PitNET (NF-PitNET), which are pituitary tumours not associated with clinical evidence of hormonal hypersecretion, represents one-third of PitNETs and three quarters of pituitary macroadenoma (4). Notably, this transcription factor-based classification is particularly practical, as NF-PitNETs often have distinct cell lineage identified after utilization of transcription factors, and up to 95% of initially hormone-negative PitNETs had positive staining with transcription factors (5). However, since the release of the latest 2022 WHO classification, its clinical application on NF-PitNETs and the implication on long term outcomes remain to be evaluated. Indeed, there has been suggestions that the updated pathological classification provides limited clinical significance (6). Therefore, we conducted this study to investigate the clinical utility of the 2022 WHO classification, as compared with the 2017 WHO classification, using a local cohort of patients with NF-PitNETs. The study examined the cross-sectional associations of these transcription factors with the clinical characteristics and radiological features, as well as their longitudinal associations with the development of long-term outcomes.

## Materials and methods

### Subject recruitment

This was a single-centre, retrospective observational study conducted at Queen Mary Hospital, Hong Kong, in accordance with the Declaration of Helsinski. The study protocol was reviewed and approved by the Institutional Review Board of the University of Hong Kong/Hospital Authority, Hong Kong West Cluster (Ref: UW 20-672). Written informed consent was waived due to the minimal risk to participants. Patients aged 18 years or above, who first presented and underwent neurosurgical resection between 1 January 2010 and 31 March 2022, and with a diagnosis of NF-PitNET were identified *via* the Clinical Data and Reporting System (CDARS) of the Hospital Authority, Hong Kong and verified with their electronic health records. In this study, exclusion criteria consisted of patients who were known germline mutation carriers of hereditary syndromes of multiple endocrine tumours. Moreover, patients whose histological specimen showed no viable pituitary tissues or insufficient tissue blocks for staining were also excluded.

### Clinical assessments

Clinical and demographic data, as well as consultation and admission notes of the included participants were retrieved and examined *via* the Clinical Management System (CMS) of the Hospital Authority, Hong Kong. Biochemical results of preoperative hormonal workup including serum levels of PRL, TSH, free thyroxine (fT4), morning adrenocorticotropic hormone (ACTH) and cortisol, follicle stimulating hormone (FSH), luteinizing hormone (LH), testosterone (in men), estradiol (in women), GH, insulin growth factor-1 (IGF-1) and alpha subunit were retrieved. The approach of pituitary surgery, operation time, as well as post-operative complications were also recorded.

### Histological analysis

For all included participants, the archival slides of their surgical specimens were retrieved and the pathological diagnosis of PitNET was confirmed by an experienced neuropathologist. Their formalin-fixed, paraffin-embedded blocks of surgical specimens were retrieved and stained for the transcription factors SF-1, TPIT and Pit-1 using the Leica BOND III fully automated *in-situ* hybridization (ISH) staining system. The following commercially available antibodies and concentrations were used: TPIT (Sigma-Aldrich, 1:200), SF-1 (R&D, 1:250), and Pit-1 (Santa Cruz, 1:200). Re-staining for Ki-67 proliferation marker (Dako, 1:250), prolactin (Dako, 1:20), FSH (BioG, 1:20), GH (NeoMarker, 1:3000), ACTH (Dako, 1:40) and/or TSH (BioG, 1:20) were also performed if not done previously. The NF-PitNETs were classified into different subgroups according to both 2017 and 2022 WHO classifications.

### Definition of clinical variables and tumour-related outcomes

In this study, the diagnosis of NF-PitNET was based on the absence of clinical and biochemical evidence of pituitary hormonal excess, including acromegaly, Cushing’s syndrome, thyrotoxicosis and significant hyperprolactinaemia suggestive of prolactinoma. The latter was defined as an elevation of serum prolactin levels higher than 2000mIU/L (7). Hypopituitarism was defined as deficiency of one or more anterior pituitary hormones with their individual hormone cut-offs determined as per the laboratory reference intervals of QMH. Central hypothyroidism was defined as the presence of low serum fT4 with normal or low serum TSH. Secondary cortisol insufficiency was defined as the presence of low serum morning cortisol with normal or low serum ACTH, or with a peak stimulated cortisol of less than 500nmol/L and with low or normal basal ACTH in 1 microgram short synacthen test (SST). Hypogonadotropic hypogonadism was defined as the presence of oligomenorrhoea or secondary amenorrhea with low serum FSH in women, or the presence of hypogonadal symptoms with low morning testosterone in men. Elevated alpha subunit level was defined as values above 0.5ng/ml in men, above 1.2ng/ml in premenopausal women and above 1.8ng/ml in postmenopausal women. Permanent cranial diabetes insipidus (DI), or arginine vasopressin (AVP) deficiency, was defined by the presence of DI beyond 14 days after the operation. Invasive tumours were defined by the presence of cavernous or sphenoidal invasion, or >50% carotid encasement by the tumour. Giant adenoma referred to tumours with any dimension that was greater than 4cm. Gross total resection of the tumour was defined as the absence of residual tumour on the first post-operative magnetic resonance imaging (MRI) (8–10).

In this study, a composite long-term tumour-related outcome, defined as the presence of residual or recurrent tumour as of 31 December 2021, was examined. Participants were considered disease-free if they did not have residual or recurrent tumours after their index operation throughout the whole observation period. Among participants with residual tumours after their index operation, stable disease referred to tumours without subsequent tumour regrowth, whereas progressive disease was defined as interval tumour enlargement during the observation period. Recurrence was defined as the reappearance of tumour on reassessment MRI scans among participants who had achieved gross total resection of tumour in their index operation. Adjuvant therapy included all forms of intervention (radiotherapy, radiosurgery or reoperation) given at any time after the index operation. In this study, in order to ensure adequate reassessment imaging had been performed during follow-up for fair evaluation of the above long-term outcomes, only participants who had two or more post-operative reassessment MRI scans performed over a follow-up duration of at least 18 months as of the aforementioned end of observation period were included in the analysis of long-term outcomes.

### Statistical analysis

In this study, all data analysis was performed using IBM SPSS Statistics Version 26.0. Normality of data was determined using Kolmogorov-Smirnov test. Results were reported as means ± standard deviation (SD), medians with inter-quartile range (IQR) or percentages, as appropriate. Independent *t*-test or Mann-Whitney U test, and one-way analysis of variance (ANOVA) or Kruskal Wallis test were used for comparisons of continuous variables between two and multiple groups, respectively, depending on the normality of data. Categorical data was compared using Chi-square or Fisher Exact test. Multivariable logistic regression analysis was used to evaluate the independent factors associated with gross total resection of NF-PitNET. Multivariable Cox regression analysis was performed to evaluate the associations of NF-PitNET of different lineages with long-term outcomes. The variables included in multivariable regression models were those that were statistically significant in the respective univariate analysis. For multivariable Cox-regression analysis, the proportional hazards assumption was checked and verified using a global goodness-of-fit test proposed by Schoenfeld. The disease-free survivals between the four lineage-restricted subtypes of NF-PitNETs were plotted by Kaplan-Meier method and compared by log-rank test. In all statistical tests, a two-sided *p*-value of <0.05 was considered as statistically significant.

## Results

**Figure 1:**
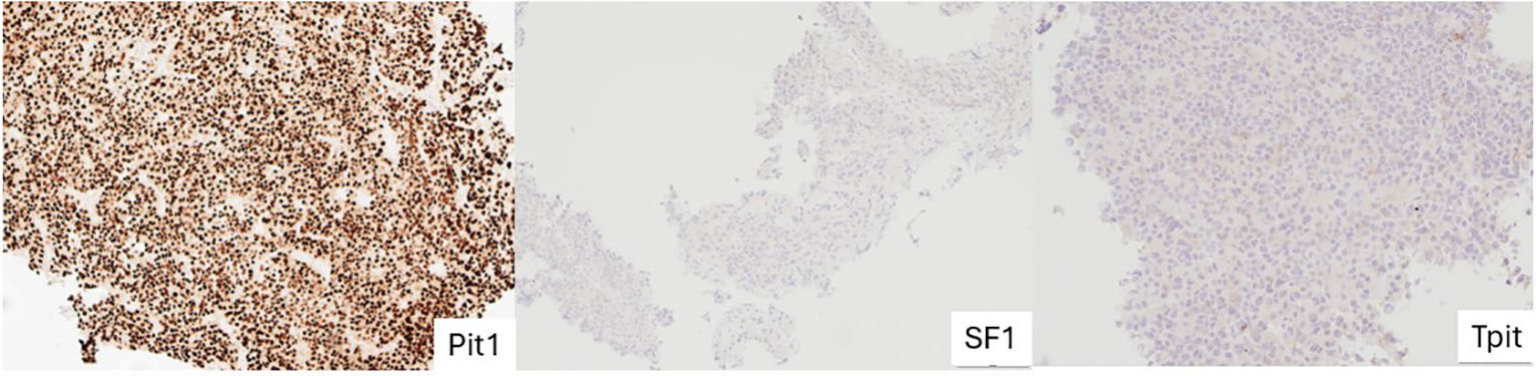
Stained images from a subject with Pit-1 lineage PitNET showing intense, uniformed, nuclear staining with Pit-1 and absent SF-1 and TPIT staining. PitNET, pituitary neuroendocrine tumour.

**Figure 2:**
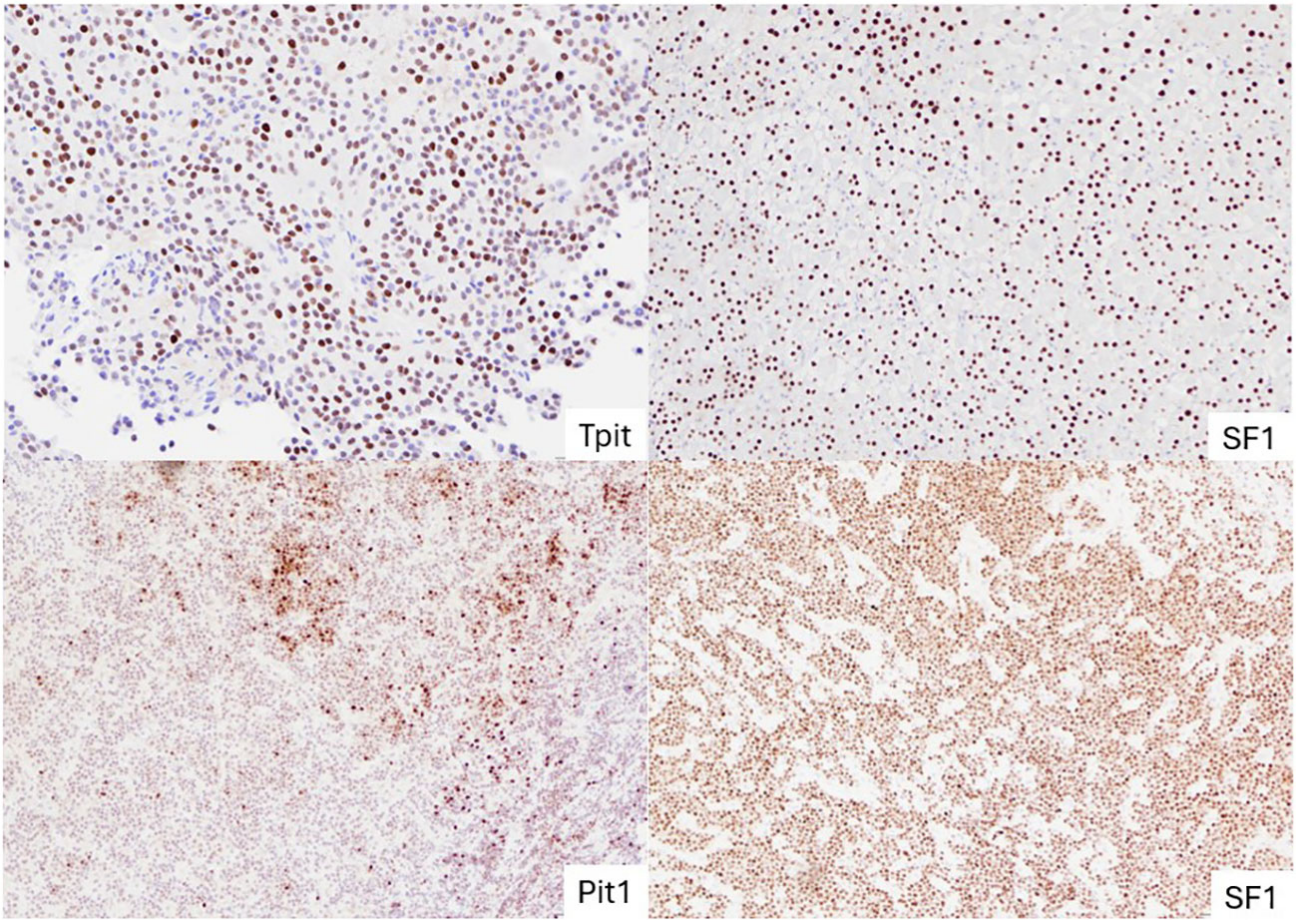
Representative images of NF-PitNETs of different lineages. TPIT-lineage PitNET with diffuse nuclear staining of TPIT (top left); SF-1 lineage PitNET with uniform nuclear staining of SF-1 (top right); PitNET without distinct lineage (subtype plurihormonal tumour) with both Pit-1 and SF-1 stained positive (bottom two). PitNET, pituitary neuroendocrine tumours.

**Figure 3:**
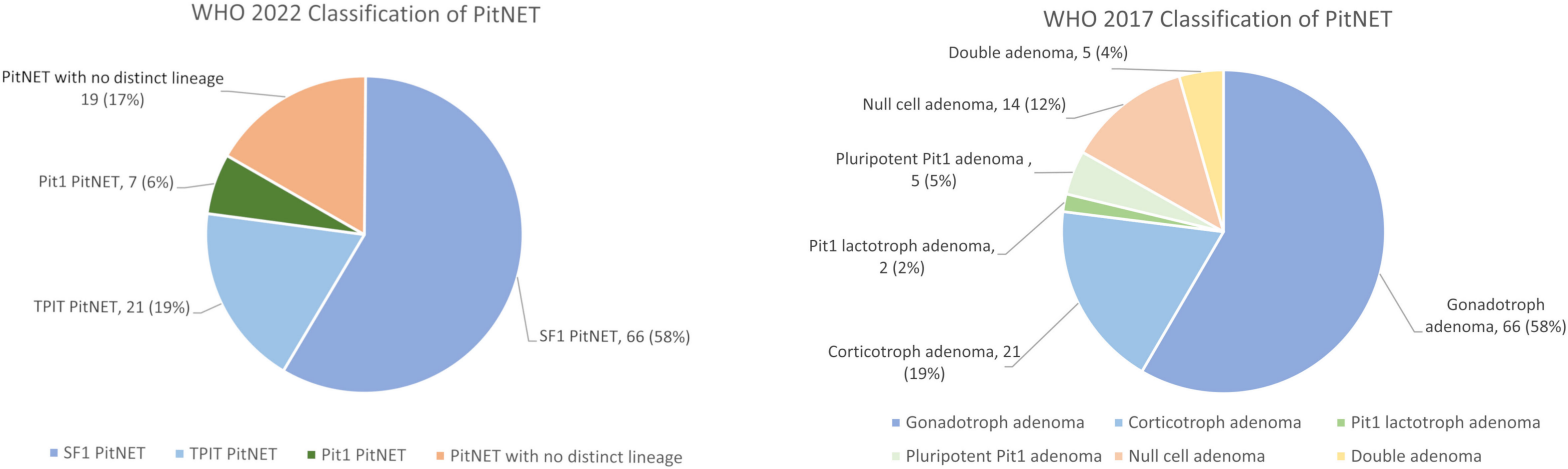
The distribution of PitNET subtypes in our cohort based on the 2022 and 2017 WHO classification. PitNET, pituitary neuroendocrine tumour; WHO, World Health Organization.

A total of 113 patients (48.7% men) with NF-PitNETs were included in this study (**Supplementary Figure 1**). Their baseline clinical characteristics, radiological and operative findings are summarized in **Supplementary Table 1**. Of these 113 NF-PitNETs, 66 were positive for SF-1 only, 21 for TPIT only, 7 for Pit-1 only, 4 were positive for both SF-1 and Pit-1, 1 was positive for both TPIT and Pit-1, while the remaining 14 were negative for all the transcription factors (**Table 1**; **Figures 1**, **2**). IHC staining for transcription factors were found to correlate poorly with that for anterior pituitary hormones. Eighty (70.8%) NF-PitNETs were considered as null cell tumours if only hormone IHC staining was used, but, after staining for transcription factors, only 9 of these 80 NF-PitNETs were also negative for all three transcription factors. Among those NF-PitNETs with negative IHC staining for pituitary hormones but were subsequently stained positive for transcription factor, the majority were SF-1-lineage PitNETs (63.8%), followed by TPIT-lineage PitNETs (18.8%). **Figure 3** illustrates the distribution of subtypes of NF-PitNET in our cohort based on the 2017 and 2022 WHO classification. When classified using the 2022 WHO classification, as compared to the 2017 classification, the proportions of SF-1-lineage (58%) and TPIT-lineage PitNETs (19%) remained the same. The 2 subjects with silent lactotroph tumours and 5 subjects with immature Pit-1-lineage tumours were re-classified under the category of Pit-1-lineage PitNETs. On the other hand, the 14 subjects with null cell adenoma and 5 subjects with pluri-hormonal tumours with different transcription factor combinations were grouped together under the entity of PitNET without distinct lineage, which then constituted 17% of our NF-PitNET cohort. **Table 2** shows the clinical and radiological features of these four subtypes of PitNET. Subjects with SF-1-lineage PitNET were significantly older compared with the other subtypes (p=0.006), while invasive tumours were more prevalent in those with TPIT-lineage PitNET (p=0.002). A numerically higher proportion of subjects with SF-1-lineage (52.4%) and Pit-1-lineage PitNETs (42.9%) achieved gross total resection than those with TPIT-lineage PitNETs (33.3%) and PitNETs without distinct cell lineage (21.1%), although there was no statistically significant difference among groups (p=0.077).

**Table 1:** Distribution of staining patterns for anterior pituitary hormones across the four lineages of NF-PitNETs under the 2022 WHO classification.

	SF-1- lineage	TPIT- lineage	Pit-1-lineage	No distinct lineage
IHC staining for hormones				
**ACTH (+)**	2	6		3
**FSH (+)**	5			
**PRL (+)**	1		2	1
**GH, TSH or multiple (+)**	7		5	1
**All negative**	51	15		14*
**Total**	66	21	7	19

ACTH, adrenocorticotropic hormone; FSH, follicular stimulating hormone; PRL, prolactin; GH, growth hormone; TSH, thyroid stimulating hormone. *Four were stained positively for both SF-1 and Pit-1, while one was stained positively for both TPIT and Pit-1.

**Table 2:** NF-PitNET subtypes and their clinico-radiological characteristics.

	SF-1-lineage (N = 66)	TPIT-lineage (N = 21)	Pit-1-lineage (N=7)	No distinct cell lineage (N=19)	p-value
Age, years	58.0 ± 12.2	51.8 ± 11.8	41.9 ± 16.8^a^	53.2 ± 13.6	**0.006**
Men	42.0 (63.6%)^b^	1.0 (4.8%)	3.0 (42.9%)	9.0 (47.4%)^b^	**<0.001**
Tumour volume, cm^3^ ^*^	16.1 ± 17.6	36.9 ± 58.1	11.5 ± 11.8	10.3 ± 5.8	0.066
Invasive tumour	20.0 (30.0%)^b^	16.0 (76.2%)	2.0 (28.6%)	7.0 (36.8%)	**0.002**
Optic nerve compression	34.0 (51.5%)	6.0 (28.6%)	2.0 (28.6%)	8.0 (42.1%)	0.237
Ki-67 index >3%	6.0 (0.1%)	0 (0%)	0 (0%)	1.0 (5.3%)	0.419
Gross total resection	33.0 (52.4%)	7 (33.3%)	3 (42.9%)	4 (21.1%)	0.077

*N=56, 19, 7, 14 with pre-op MRI available for subjects who had NF-PitNET with SF-1, TPIT, Pit-1-lineage and without distinct cell lineage, respectively. ^a^p<0.05 (SF-1-lineage as referent); ^b^ p<0.05 (TPIT-lineage as referent).

**Figure 4:**
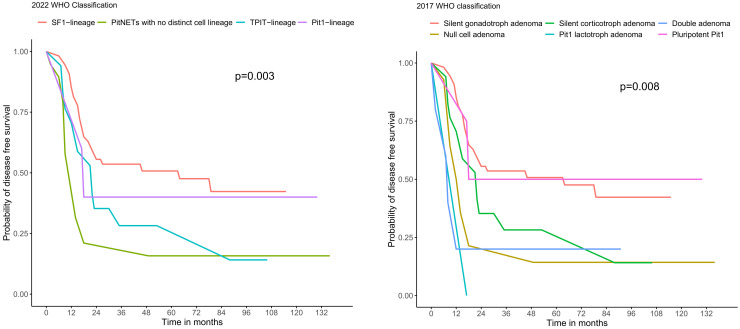
Kaplan-Meier curves showing the disease-free survival of the subtypes of NF-PitNET based on 2022 and 2017 WHO classification. PitNET, pituitary neuroendocrine tumour; WHO, World Health Organization.

Among these 113 subjects, 95 of them had two or more post-operative MRI scans available and hence were included for evaluation of their long-term outcomes. There were no significant differences in age, sex, tumour size, resection status and post-operative hormonal replacement requirements between these 95 subjects and those not included in the long-term outcome analysis. Over a median follow-up of 5.7 years (IQR 2.8 – 8.3), there were significant differences in the disease-free survival amongst the four subtypes of NF-PitNET (Log-rank test p=0.003) (**Figure 4**). Significant survival difference was present between subjects with SF-1-lineage tumours and those with PitNET without distinct lineage (Log-rank test p=<0.001). On the other hand, when using the 2017 WHO classification, corresponding differences in disease-free survival was also demonstrated amongst the different subtypes of NF-PitNET (Log-rank test p=0.008) (**Figure 4**). Consistently, the difference in disease-free survival was significant between gonadotroph adenoma and null cell adenoma (Log-rank test p=0.001), as well as between gonadotroph adenoma and double adenoma (Log-rank test p=0.01). The recurrence rate was numerically the highest among TPIT-lineage PitNETs (17.6%), followed by PitNETs without distinct lineage (5.3%), SF-1 lineage (3.7%) and Pit-1 lineage PitNETs (0%), although differences between groups did not reach statistical significance (p=0.053) (**Supplementary Table 2**). In multivariable Cox regression analysis, both tumour volume (HR 1.04, 95% CI 1.01-1.07, p=0.017) and the subtype of PitNET without distinct lineage (HR 3.02, 95% CI 1.28-7.16, p=0.012) were independently associated with the development of residual or recurrent disease (**Table 3**).

**Table 3:** Cox regression analyses showing the associations of clinical, radiological and pathological characteristics with the composite long-term outcome of residual or recurrent disease.

	Univariate analysis		Multivariable analysis	
	HR (95%CI)	*p*-value	HR (95%CI)	*p*-value
Age, years	0.99 (0.97-1.01)	0.504	–	–
Men	0.66 (0.40-1.10)	0.111	–	–
Tumour volume, cm^3^	1.01 (1.003-1.018)	**0.008**	1.04 (1.002-1.070)	**0.017**
Tumour invasiveness	1.80 (1.08-3.00)	**0.023**	1.11 (0.59-2.06)	0.750
PitNET subtypes		**0.005**		**0.007**
SF-1-lineage	Referent	–	Referent	–
TPIT-lineage	1.81 (0.93-3.49)	0.079	1.29 (0.58-2.84)	0.533
Pit-1-lineage	1.07 (0.33-3.54)	0.909	1.10 (0.33-2.84)	0.881
No distinct cell lineage	3.01 (1.61-5.56)	**0.001**	3.44 (1.70-6.96)	**<0.001**

HR, hazard ratio; 95%CI, 95% confidence interval; PitNET, pituitary neuroendocrine tumour.

## Discussions

The role of transcription factor in the classification of PitNET had been shown to be essential in various studies since the introduction of the WHO 2017 classification (5, 11–13). The three key transcription factors SF-1, TPIT and Pit-1, provided a new doctrine that not only allows higher diagnostic accuracy but also demonstrates prognostic correlation, which previous classifications based on IHC staining of anterior pituitary hormones have not been able to achieve. While there has been controversies about the clinical significance of this new classification (6), our current study of NF-PitNETs demonstrated that, as compared to previous classifications, the latest WHO 2022 classification provided a simpler and more straight-forward classification comprising only four major entities of PitNETs and was still able to demonstrate different courses of long-term outcomes, specifically in the disease-free survival between SF-1-lineage PitNET and PitNET without distinct lineage.

When comparing the 2022 and 2017 WHO classification, the prevalence of silent gonadotroph adenoma (based on 2017) or SF-1 lineage PitNET (based on 2022), as well as silent corticotroph adenoma (based on 2017) or TPIT lineage PitNET (based on 2022), remained unchanged. The new category of PitNET without distinct lineage, which was first introduced in WHO 2022 classification, combines previous entities of plurihormonal PitNET and null cell PitNET (2). In our study, this new entity became the third most common subtype under the new classification. Moreover, with regard to prognosis, in our subjects, when using the 2017 WHO classification, those with null cell adenoma and double adenoma had less disease-free survival as compared with gonadotroph adenoma. Consistently, our subjects with PitNET without distinct lineage were observed to behave in a more aggressive manner with less disease-free survival than SF-1-lineage PitNETs. Indeed, previous studies have shown that null cell adenoma was a more aggressive subtype that was prone to invade with a higher risk of residual disease (12, 14). Double adenoma, also known as multiple pituitary adenoma, is an uncommon entity with reported prevalence of 0.1-2.6%, with majority of them presenting as functioning tumours. The most common patterns of multiple pituitary adenomas are PIT-1-lineage combined with SF-1-lineage, followed by PIT-1-lineage combined with TPIT-lineage (15), which were in keeping with our findings. Scarce evidence is available regarding prognosis of double adenoma due to its rarity, but some reviews suggested a higher chance of surgical failure due to lack of awareness and incomplete resection (16, 17). By unifying these entities without distinct lineage, the 2022 WHO classification facilitates the identification of a PitNET subtype with a less favourable prognosis.

Despite the merits of the current transcription factor-based classification, several pitfalls remain. First, IHC stain interpretation is a semi-quantitative method that relies on skills and experience of histopathologists, and interpersonal variability may occur (18). Moreover, pituitary tumorigenesis is a complex process that cannot be simply explained by three transcription factors alone. Interplay between transcription factors, variability of protein expression, as well as trans-differentiation of tumours is still not well understood (19). Whether tumours with expression of two transcription factors without immunostaining of corresponding hormones should be regarded as a plurihormonal PitNET, and strategies to overcome incongruent expression of IHC of transcription factors and hormones, remained to be elucidated in further studies (6, 19).

Indeed, the availability of sensitive and specific antibodies against transcription factors for IHC staining is important. False-positive SF-1 staining with some anti-SF-1 antibodies has been reported (6, 20). Moreover, aborted protein expression of a specific gene can influence the interpretation of the true lineage of PitNET. The use of RNAscope, an RNA *in-situ* hybridization technique which utilizes visualization of single mRNA molecule in individual cells, might help to resolve these issues. Some recent studies employing RNAscope found mRNA expression of programmed death ligand 1 (PD-L1) and cytotoxic T lymphocyte-associated protein 4 (CTLA4) in pituitary adenomas (21, 22).

A major limitation of the current WHO classification of PitNET is that it relies on tumour histotype as the sole prognostic marker for PitNET without incorporating any grading or stratification system (19). Evidence suggests that the biological determinants of aggressive PitNET is not only based solely on transcription factor subtype, but also relies on an interplay of different proliferative, radiological and molecular factors. Trouillas et al. advocated a grading system incorporating radiological invasiveness, as well as comprehensive cell cycle proliferative markers that correlate with long-term disease-free and recurrence/progression-free status, which has been validated in several series (23–26). In addition, while a few recent novel molecular markers such as analysis of PitNET microRNA (miRNA) expression profiles including Ubiquitin Specific Peptidase 8 (USP8) mutational status, DNA methylation status and expressions of VEGF-A/VEGFR have been shown to correlate with PitNET invasiveness and aggressiveness in small studies, they remain investigational and routine usage are still limited by availability (27). Future editions of WHO classification can be strengthened *via* further integration of clinical, radiological and pathological grading (28).

Our study has several limitations, which included the retrospective study design and the use of archived surgical specimens that could have influenced the immunogenicity of the IHC stain. Secondly, the determination of the subtypes of PitNET was interpreted by one single pathologist and routine staining for the transcription factors was relatively new in our centre. Moreover, our study had a relatively small sample size, especially in the longitudinal analysis of long-term outcomes. Nonetheless, our study still showed significant differences in disease-free survival between the subtypes of NF-PitNET. Further studies that include a larger sample size and longer duration of follow-up would be beneficial in confirming findings of our study and identifying additional predictors of aggressiveness in PitNET. In addition, null cell adenoma is defined as the lack of all transcription factors staining in our study (12), as opposed to some other studies which applied a more stringent definition of lacking both transcription factors as well as hormone IHC staining (5). There were five null cell adenomas that were stained negative for all transcription factors which also exhibited weak and focal hormone IHC staining (three ACTH only, one ACTH with concomitant prolactin and one prolactin only). After excluding these four entities from the subtype of PitNET without distinct lineage, sensitivity analysis shows similar findings as the results concluded in our study.

## Conclusion

This study on NF-PitNETs demonstrated the clinical utility of the WHO 2022 classification, which can be considered as a simplified version of its 2017 predecessor with fewer entities, and may be more convenient for clinical application and comprehension. Our findings also highlighted the importance of subtyping PitNET for prognostic stratification, which allows a more personalized clinical management, especially in post-operative monitoring and surveillance for patients with PitNETs.

## Data availability statement

The original contributions presented in the study are included in the article/**Supplementary Material**. Further inquiries can be directed to the corresponding author.

## Ethics statement

The studies involving humans were approved by Institutional Review Board of the University of Hong Kong/Hospital Authority, Hong Kong West Cluster, Hong Kong. The studies were conducted in accordance with the local legislation and institutional requirements. The ethics committee/institutional review board waived the requirement of written informed consent for participation from the participants or the participants’ legal guardians/next of kin because the study involved minimal risk to participants.

## Author contributions

CW: Conceptualization, Formal analysis, Methodology, Project administration, Writing – original draft, Writing – review & editing, Visualization. RH: Writing – original draft, Writing – review & editing, Validation. GH: Writing – original draft, Writing – review & editing, Validation. H-TL: Writing – original draft, Writing – review & editing, Validation. CF: Formal analysis, Writing – original draft, Writing – review & editing. JC: Writing – original draft, Writing – review & editing. EL: Writing – original draft, Writing – review & editing. LT: Writing – original draft, Writing – review & editing. IM: Writing – original draft, Writing – review & editing. AL: Writing – original draft, Writing – review & editing. DL: Writing – original draft, Writing – review & editing. Y-CW: Writing – original draft, Writing – review & editing. W-SC: Writing – original draft, Writing – review & editing. GL: Writing – original draft, Writing – review & editing. KT: Writing – original draft, Writing – review & editing. KL: Writing – original draft, Writing – review & editing. C-HL: Conceptualization, Project administration, Supervision, Writing – original draft, Writing – review & editing, Methodology.

## References

[1] Lloyd RV OR Klöppel G Rosai J. WHO classification of tumours of endocrine organs. France: IARC Publications. (2017).

[2] Asa SL Mete O Perry A Osamura RY. Overview of the 2022 WHO classification of pituitary tumors. Endocrine Pathol. (2022) 33(1):6–26. doi: 10.1007/s12022-022-09703-7 35291028

[3] Inoshita N Nishioka H. The 2017 WHO classification of pituitary adenoma: overview and comments. Brain Tumor Pathol. (2018) 35:51–6. doi: 10.1007/s10014-018-0314-3 29687298

[4] AlMalki MH Ahmad MM Brema I AlDahmani KM Pervez N Al-Dandan S. Contemporary management of clinically non-functioning pituitary adenomas: A clinical review. Clin Med Insights: Endocrinol Diabetes. (2020) 13:1–13. doi: 10.1177/1179551420932921 PMC7318824 32636692

[5] Nishioka H Inoshita N Mete O Asa SL Hayashi K Takeshita A. The complementary role of transcription factors in the accurate diagnosis of clinically nonfunctioning pituitary adenomas. Endocr Pathol. (2015) 26(4):349–55. doi: 10.1007/s12022-015-9398-z 26481628

[6] Wan X-y Chen J Wang J-w Liu YC Shu K Lel T. Overview of the 2022 WHO classification of pituitary adenomas/pituitary neuroendocrine tumors: clinical practices, controversies, and perspectives. Curr Med Sci. (2022) 42:1111–8. doi: 10.1007/s11596-022-2673-6 36544040

[7] Karavitaki N Thanabalasingham G Shore HCA Trifanescu R Ansorge O Meston N Turner HE Wass JAH. Do the limits of serum prolactin in disconnection hyperprolactinaemia need re-definition? A study of 226 patients with histologically verified non-functioning pituitary macroadenoma. Clin Endocrinol. (2006) 65(4):524–9. doi: 10.1111/j.1365-2265.2006.02627.x 16984247

[8] Cottier J-P Destrieux C Brunereau L Moreau PBL Jan M Herbreteau D. Cavernous sinus invasion by pituitary adenoma: MR imaging. Radiology. (2000) 215:264–9. doi: 10.1148/radiology.215.2.r00ap18463 10796926

[9] Ieva AD Rotondo F Syro LV Kovacs MDCK. Aggressive pituitary adenomas—diagnosis and emerging treatments. Nat Rev. (2014) 10(7):423–35. doi: 10.1038/nrendo.2014.64 24821329

[10] Micko ASG Wöhrer A Wolfsberger S Knosp E. Invasion of the cavernous sinus space in pituitary adenomas: endoscopic verification and its correlation with an MRI-based classification. J Neurosurg. (2015) 122(4):803–11. doi: 10.3171/2014.12.JNS141083 25658782

[11] Liu J He Y Zhang X Yan X Huang Y. Clinicopathological analysis of 250 cases of pituitary adenoma under the new WHO classification. Oncol Lett. (2020) 19:1890–8. doi: 10.3892/ol PMC7039149 32194684

[12] Hong SW Kim SH Lim SH Lee EJ Kim SH Ku CR. Clinical relevance of new world health organization classification system for pituitary adenomas: A validation study with 2-year experience. Front Oncol. (2021) 11. doi: 10.3389/fonc.2021.739290 PMC8476031 34589436

[13] Silva-Ortega S García-Martinez A Jaime M Torregrosa ME Abarca J Monjas I. Proposal of a clinically relevant working classification of pituitary neuroendocrine tumors based on pituitary transcription factors. Hum Pathol. (2021) 110:20–30. doi: 10.1016/j.humpath.2020.12.001 33321163

[14] Balogun JA Monsalves E Juraschka K Parvez K Kucharczyk W Mete O. Null cell adenomas of the pituitary gland: an institutional review of their clinical imaging and behavioral characteristics. Endocr Pathol. (2015) 26:63–70. doi: 10.1007/s12022-014-9347-2 25403448

[15] Budan RM Georgescu CE. Multiple pituitary adenomas: A systematic review. Front Endocrinol. (2016) 7. doi: 10.3389/fendo.2016.00001 PMC4740733 26869991

[16] Zieliński G Sajjad EA Maksymowicz M Pękul M Koziarski A. Double pituitary adenomas in a large surgical series. Pitutiary. (2019) 22:620–32. doi: 10.1007/s11102-019-00996-2 PMC6842358 31598814

[17] Ogando-Rivas E Alalade AF Boatey J Schwartz TH. Double pituitary adenomas are most commonly associated with GH- and ACTH-secreting tumors: systematic review of the literature. Pitutiary. (2017) 20:702–8. doi: 10.1007/s11102-017-0826-6 28766078

[18] García-Martínez A Johana Sottile CF Pedro Riesgo RC Juan Antonio Simal CL Sandoval H Aranda I. Is it time to consider the expression of specific pituitary hormone genes when typifying pituitary tumours? PloS One. (2018) 13(7):e0198877. doi: 10.1371/journal.pone.0198877 29979686 PMC6034784

[19] Villa C Baussart B Assié G Raverot G Roncaroli F. The World Health Organization classifications of pituitary neuroendocrine tumours: a clinico-pathological appraisal. Endocrine-Related Cancer. (2023) 30(8):e230021. doi: 10.1530/ERC-23-0021 37068095

[20] McDonald WC Banerji N McDonald KN Ho B Macias V Kajdacsy-Balla A. Steroidogenic factor 1, pit-1, and adrenocorticotropic hormone A rational starting place for the immunohistochemical characterization of pituitary adenoma. Arch Pathol Lab Med. (2017) 141:104–12. doi: 10.5858/arpa.2016-0082-OA 27227698

[21] Mei Y Bi WL Greenwald NF Du Z Agar NYR Kaiser UB. Increased expression of programmed death ligand 1 (PD-L1) in human pituitary tumors. Oncotarget. (2016) 7:76565–76. doi: 10.18632/oncotarget.v7i47 PMC5363530 27655724

[22] Sabini E Khan A Caturegli P. Сytotoxic T lymphocyte-associated protein 4 (CTLA4) is overexpressed in a subset of prolactin- and growth hormone-secreting pituitary adenomas. Endocrine-Related Cancer. (2024) 31(1):e230196. doi: 10.1530/ERC-23-0196 PMC11249045 37870923

[23] Trouillas J Roy P Sturm N Dantony E Cortet-Rudelli C Viennet G. A new prognostic clinicopathological classification of pituitary adenomas: a multicentric case–control study of 410 patients with 8 years post-operative follow-up. Acta Neuropathol. (2013) 126:123–35. doi: 10.1007/s00401-013-1084-y 23400299

[24] Asioli ARS Iommi M Baldovini C Ambrosi F Guaraldi F Zoli M. Validation of a clinicopathological score for the prediction of post-surgical evolution of pituitary adenoma: retrospective analysis on 566 patients from a tertiary care centre. Eur J Endocrinol. (2019) 180:127–34. doi: 10.1530/EJE-18-0749 30481158

[25] Raverot G Beauvy EDJ Vasiljevic A Mikolasek S Borson-Chazot F Jouanneau E. Risk of recurrence in pituitary neuroendocrine tumors: A prospective study using a five-tiered classification. J Clin Endocrinol Metab. (2017) 102:3368–74. doi: 10.1210/jc.2017-00773 28651368

[26] Lelotte J Mourin A Fomekong E Michotte A Raftopoulos C Maiter D. Both invasiveness and proliferation criteria predict recurrence of non-functioning pituitary macroadenomas after surgery: a retrospective analysis of a monocentric cohort of 120 patients. Eur J Endocrinol. (2018) 178(3):237–46. doi: 10.1530/EJE-17-0965 29259039

[27] Bioletto F Berton AM Prencipe N Varaldo E Bona C Grottoli S. Markers of aggressiveness in pituitary tumors: update and perspectives. J Clin Med. (2022) 11(21):6508. doi: 10.3390/jcm11216508 36362740 PMC9658464

[28] Guaraldi F Zoli M Righi A Gibertoni D Picciola VM Faustini-Fustini M. A practical algorithm to predict postsurgical recurrence and progression of pituitary neuroendocrine tumours (PitNET)s. Clin Endocrinol. (2020) 93:36–43. doi: 10.1111/cen.14197 32306401

