# Supplementary material for: A clinicopathological study of non-functioning pituitary neuroendocrine tumours using the World Health Organization 2022 classification
Source: Front Endocrinol (Lausanne). 2024 May 2;15:1368944. doi: 10.3389/fendo.2024.1368944 (PMC11096571; doi:10.3389/fendo.2024.1368944)
Supplement: Supplementary file 1 [file DataSheet_1.docx]

Supplementary Table 1 Clinical characteristics and radiological features of subjects with NF-PitNET in the cohort

| Clinical variables |  |
| --- | --- |
| Age, years | 54.8±13.5 |
| Men | 55.0 (48.7%) |
| Smoking status* |  |
| Never smoker | 77.0 (81.1%) |
| Ex-smoker | 12.0 (12.6%) |
| Current smoker | 6.0 (6.3%) |
|  |  |
| Clinical presentation |  |
| Blurring of vision | 66.0 (58.4%) |
| Headache | 30.0 (26.5%) |
| Symptoms of hypopituitarism | 29.0 (25.7%) |
| Incidental finding on brain imaging | 26.0 (23.0%) |
|  |  |
| Pre-operative hormonal profile† |  |
| Hypogonadotropic hypogonadism | 56.0 (53.3%) |
| Hyperprolactinaemia | 26.0 (23.6%) |
| Secondary cortisol insufficiency | 16.0 (14.8%) |
| Elevated alpha subunit | 11.0 (11.7%) |
| Central hypothyroidism | 8.0 (7.5%) |
|  |  |
| Radiological features# |  |
| Median largest dimension of tumour, cm | 2.70 (2.25-3.25) |
| Largest dimension >4cm | 10.0 (9.5%) |
| Tumour invasiveness | 45.0 (39.8%) |
| Optic chiasmal compression | 50.0 (44.2%) |
|  |  |
| Operative features |  |
| Approach |  |
| Transsphenoidal | 102.0 (90.3%) |
| Combined | 9.0 (8.0%) |
| Open brain | 2.0 (1.8%) |
| Post-operative serum sodium disturbance | 65.0 (57.5%) |
| Transient AVP deficiency | 20.0 (30.8%) |
| Transient SIADH | 18.0 (27.7%) |
| Permanent AVP deficiency | 10.0 (15.4%) |
| Triphasic | 9.0 (13.8%) |

Data presented as N (%), mean ± standard deviation or median (interquartile range). NF-PitNET, non-functioning pituitary neuroendocrine tumour; AVP, arginine vasopression; SIADH, syndrome of inappropriate antidiuretic hormone. *N=95 for subjects with available data on their smoking status; †N=104 for subjects with full hormonal profile evaluation available including gonadal hormones, prolactin, cortisol and ACTH, fT4 and TSH, respectively, 93 subjects had alpha subunit level available; # N=105, 113 and 53 for subjects with available data on their tumour size, tumour invasiveness and MRI images for Knosp grading, respectively.

Supplementary Table 2 Individual long-term outcomes across the four subtypes of PitNETs

|  | SF1-lineage  (N = 54) | TPIT-lineage  (N = 17) | Pit1-lineage  (N=5) | | PitNETs without distinct cell lineage  (N=19) | p-value* |
| --- | --- | --- | --- | --- | --- | --- |
| Recurrence | 2.0 (3.7%) | 3.0 (17.6%) | 0 (0%) | | 1.0 (5.3%) | 0.053 |
| Need of adjuvant therapy | 10.0 (18.5%) | 7.0 (33.3%) | | 0 (0%) | 5.0 (26.3%) | 0.623 |

*Log-rank test


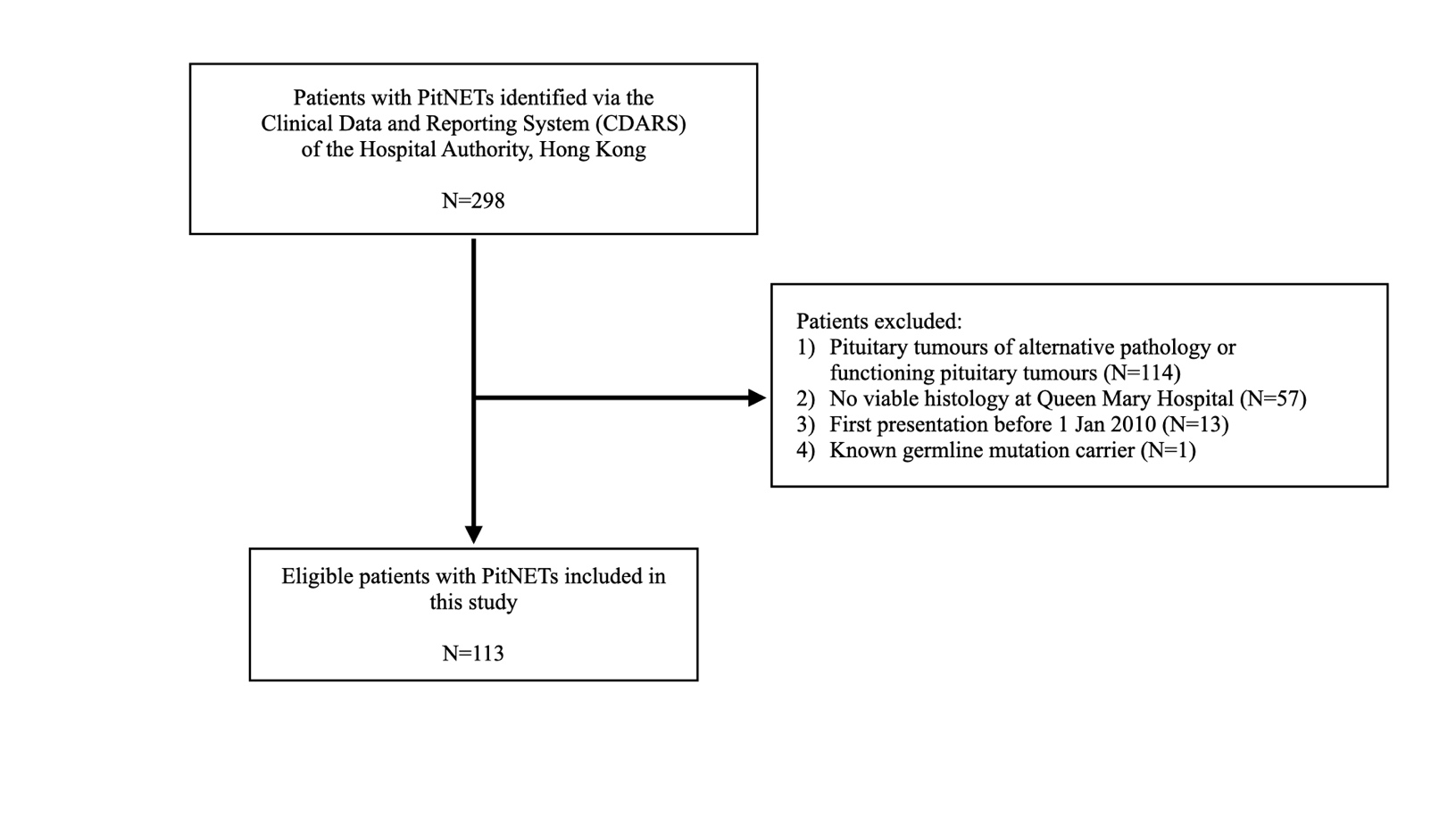
Supplementary Figure 1 Study flow diagram

PitNET, pituitary neuroendocrine tumours
